# Transition from pediatric to adult care for adolescents living with HIV in South Africa: A natural experiment and survival analysis

**DOI:** 10.1371/journal.pone.0240918

**Published:** 2020-10-27

**Authors:** Brian C. Zanoni, Moherndran Archary, Thobekile Sibaya, Nicholas Musinguzi, Jessica E. Haberer

**Affiliations:** 1 Emory University, Atlanta, Georgia, United States of America; 2 Massachusetts General Hospital, Boston, Massachusetts, United States of America; 3 Harvard Medical School, Boston, Massachusetts, United States of America; 4 University of KwaZulu-Natal Nelson Mandela School of Medicine, Durban, South Africa; 5 Mbarara University of Science and Technology, Mbarara, Uganda; University of the Witwatersrand, SOUTH AFRICA

## Abstract

**Objective:**

To determine rates of retention and viral suppression among adolescents living with perinatally-acquired HIV who remained in pediatric care compared to those who transitioned to adult care.

**Methods:**

We evaluated a natural experiment involving adolescents living with perinatally-acquired HIV who were attending a government-supported antiretroviral clinic in KwaZulu-Natal, South Africa. Prior to 2011, all adolescents transitioned to adult care at 12 years of age. Due to a policy change, all adolescents were retained in pediatric care after 2011. We analyzed adolescents two years before and two years after this policy change. Outcomes were retention in care and HIV viral suppression one year after transition to adult care or the 13^th^ birthday if remaining in pediatric care.

**Results:**

In the natural experiment, 180 adolescents who turned 12 years old between 2011 and 2014 were evaluated; 35 (20%) transitioned to adult care under the old policy and 145 (80%) remained in pediatric care under the new policy. Adolescents who transitioned to the adult clinic had lower rates of retention in care (49%; 17/35) compared to adolescents remaining in the pediatric clinic (92%; 134/145; p<0.001). Retention in care was lower (ARR 0.59; 95%CI 0.43–0.82; p = 0.001) and viral suppression was similar (ARR = 1.06, 95%CI 0.89–1.26; p = 0.53) for adolescents who transitioned to adult care compared to adolescents remaining in pediatric care.

**Conclusion:**

Adolescents living with perinatally-acquired HIV appear to have higher retention in care when cared for in pediatric clinics compared to adult clinics. Longer-term follow-up is needed to fully assess viral suppression.

## Introduction

Improvement in antiretroviral therapy (ART) has changed perinatal HIV infection from a terminal disease into a chronic, manageable infection requiring long-term care [1]. In South Africa, ART was not available until 2004, which contributed to more than 400,000 mother-to-child HIV transmissions in the early and mid 2000s [2–5]. With increasing availability of ART since then, many children living with perinatally-acquired HIV are surviving into adolescence and early adulthood; however, long-term clinical outcomes are often poor [6]. Less than 50% of adolescents living with HIV in South Africa are virally suppressed contributing to their high mortality rates [7, 8].

Adolescents living with perinatally-acquired HIV eventually require transition from pediatric to adult care [1, 2]. As the wave of perinatally HIV-infected adolescents matures, an estimated 320,000 adolescents will transfer from pediatric- or adolescent-based clinics to adult services by 2028 in South Africa alone [8, 9]. As with other chronic illnesses [10–12], this transition to adult services has been associated with poor retention in care and poor clinical outcomes for adolescents living with HIV in many settings [13–18]. In South Africa, for example, Davies et al. found that older adolescents (>15 years old) had significantly lower viral suppression and retention in care than younger adolescents at one and two years after transfer to local clinics [19]. These poor outcomes highlight the need to study and support transition care for adolescents living with HIV.

A policy change in a South African clinic afforded an informative opportunity to assess the effects of the transition to adult care. In this policy, children who had been transferred uniformly to the adult clinic at age 12 were abruptly retained in the pediatric clinic. Leveraging this natural experiment, we compared rates of short- and long-term retention in care and viral suppression among those who transitioned to adult care versus to those remaining in the pediatric clinic after the policy change.

## Methods

### Setting

Mahatma Gandhi Memorial Hospital is a regional/district hospital located in the township of KwaMashu outside of Durban, South Africa. The outpatient clinic provides care for more than 650 children living with perinatally-acquired HIV and receiving ART. The adult clinic provides care for more than 1,700 adults living with HIV and receiving ART. All care and medication are provided free of charge.

In the pediatric clinic, adolescents were seen in monthly appointments on the same day of the week each month by the same physician. Adolescents had the option to attend an unstructured support group meeting during their clinic day. During their visit, they were evaluated by a clinician and collected medication at an onsite pharmacy. In the adult clinic, adolescents were seen every three months during clinics that ran five days a week [20]. They were seen by different clinicians at each visit and collected medication at onsite pharmacy monthly. There were no support groups or additional services available in the adult clinic.

### Study design

Prior to 2011, adolescents transition*e*d to adult care if they were 12 years of age and disclosed of their HIV status (“old policy”); they received no preparation for transitioning to the adult clinic. Due to a change in policy in 2011, adolescents were subsequently retained in pediatric care (“new policy”) and not transitioned to adult care. Leveraging this natural experiment, we analyzed adolescents who turned age 12 years old and transitioned to adult care in the two year prior to the policy change (2009–2010) and those who turned age 12 in the two years following the policy change (2011–2012) who remained in pediatric care. Adolescents contributed data from the time they turned 12 years old (between 2009 to 2012) until November 15, 2016. We considered transition to adult care as the exposure; therefore, those adolescents who remained in pediatric care after their 12^th^ birthday prior to the policy change (15) were analyzed as remaining in pediatric clinic and adolescents who transitioned to the adult clinic after the policy change were analyzed as adult clinic (2). All children qualified for and initiated ART prior to age 12 years. This study design allows for the evaluation of similar cohorts with minimal difference in chronological time, avoiding changes in care delivery over time and other unmeasured confounders. By considering transition as the exposure, our approach also allows for any delays in policy implementation.

Data Collection: All adolescents and young adults who were not retained in care were tracked by phone call, home visit, or a National Department of Health Laboratory database (NHLS) search to ascertain their current retention status and to evaluate for undocumented (silent) transfers to alternate clinics [21].

### Definitions for short-term outcomes

Using a quasi-experimental design, we compared rates of retention in care and viral suppression among adolescents who remained in pediatric care compared to those who transitioned to adult care.

- Retention in care: To account for all data relevant to retention, retention in care was defined as alive patients with either a <2-month gap in pharmacy refill, or the presence of at least one clinic visit, CD4 or viral load result within 18 months from their 12^th^ birthday. The 18-month time period was chosen because national guidelines recommended viral load monitoring every 12 months and we allowed for a 6-month window period to account for late visits or missed blood draws [20]. Transfers to alternate clinics were considered retained in care if they had a clinic visit, ART dispensed elsewhere, or a viral load within 18 months after the documented transfer per the NHLS database. Silent transfers were defined as adolescents who were found at other clinics by viral load results or ART dispensing records by review of the NHLS database. Silent transfers were included in this analysis because they were in care at alternate facilities and not lost to follow-up.
- Viral suppression: For short-term outcomes, viral suppression was defined as viral load <400 copies/ml one year (+/- 6-month window period) after transition for those who transitioned to adult care or <400copies/ml after their 13^th^ birthday (+/- 6 month window period) for those remaining in pediatric care. Most recent viral load was the latest viral load for those remaining in care from Jan 1, 2015-Nov 15, 2016 (or 3–4 years after the policy change), signifying long-term outcomes. Missing viral load data or latest viral load falling before Jan 1, 2015 were considered as not suppressed indicating the last viral load falling outside of the 18-month time period [20]. Adolescents and young adults who died were considered not retained in care and not virally suppressed. We also analyzed the combined outcome of those retained and virally suppressed.

### Definitions for long-term outcomes

To look at long-term outcomes, we conducted a survival analysis in which adolescents were followed beginning at age 12 years old (between 2009 and 2012) and followed until the end of data collection, November 15, 2016. Adolescents who transferred to alternate clinics were censored at their last visit. Retention in care was defined as alive patients, <2-month gap in pharmacy refill, presence of at least one clinic visit, CD4 or viral load result within 18 months [20]. Those who were not retained in care were considered lost to follow-up on the date of their last known clinic visit or blood draw. This broad definition of retention in care is required to account for different routine follow-up intervals in pediatric compared to adult clinics.

### Data collection

We obtained demographic data, pre-ART laboratory data, ART history, pharmacy refill data, clinic visits, tuberculosis history, CD4 count, and viral load from electronic and paper medical records. A subgroup of adolescents participated in in-depth interviews enquiring about facilitators and barriers to transition care that is currently under review in a separate manuscript.

### Statistical analysis

We used descriptive statistics to understand retention status of adolescents in the two clinics under the natural experiment. We summarized demographics using medians in the case of continuous variables, and frequencies and percentages for categorical variables. Comparison of our main predictor variables by demographic and other characteristics was conducted using the Wilcoxon rank-sum test for continuous variables and Fisher’s Exact test for categorical variables. To estimate risk ratios, we fit modified Poisson generalized linear models with robust standard errors, [22] which included the main predictor (pediatric vs adult clinic) and potential confounders (sex, ART regimen, and pre-ART CD4). In addition, we performed a survival analysis using a cox proportional hazards to assess time to loss to follow-up among the adolescents in the natural experiment who had transitioned to adult care compared to those who remained in pediatric care. Time to loss to follow-up is reported using Kaplan-Meier estimates.

### Ethics statement

The Durban University of Technology Institutional Research Ethics Committee, KwaZulu-Natal Department of Health and the Partners HealthCare/Massachusetts General Hospital Research Ethics Board approved this protocol.

## Results

### Baseline characteristics

For the natural experiment, 180 adolescents turned 12 years old in the time period two years before and two years after the policy change (i.e., between 2011 and 2014). As shown in **Table 1**, 35 (19%) adolescents transitioned to adult care under the old policy and 145 (81%) adolescents remained in pediatric care under the new policy.

**Table 1:** Characteristics of adolescents who turned age 12 between 2011 and 2014 and transitioned to adult care under the old policy or remained in pediatric care under the new policy.

	Pediatric clinic (n = 145)	Transitioned to adult clinic (n = 35)
**Female (n)**	69 (48%)	21 (60%)
** pre-ART CD4 cells/mm^3^ (IQR)**	335 (130–564)	313 (153–630)
**Median log_10_ pre-ART viral load (copies/ml)**	5.2 (4.5–5.7)	4.5 (4.1–4.8)
ART at time of transition: NNRTI (n)*	124 (86%)	27 (79%)
**Years from HIV diagnosis to ART initiation (IQR)**	0.3 (0.1–0.7)	0.2 (0.1–0.5)
**Median years on ART (IQR)**	7.0 (5.5–9.2)	7.4 (5.6–9.5)
**History of tuberculosis (n)**	84 (58%)	19 (54%)

Abbreviations: IQR–interquartile range; ART–antiretroviral therapy; NNRTI–Non-nucleoside Reverse Transcriptase Inhibitor

*Those not taking NNRTI regimens received protease inhibitor therapy

### Primary outcomes

**Fig 1:**
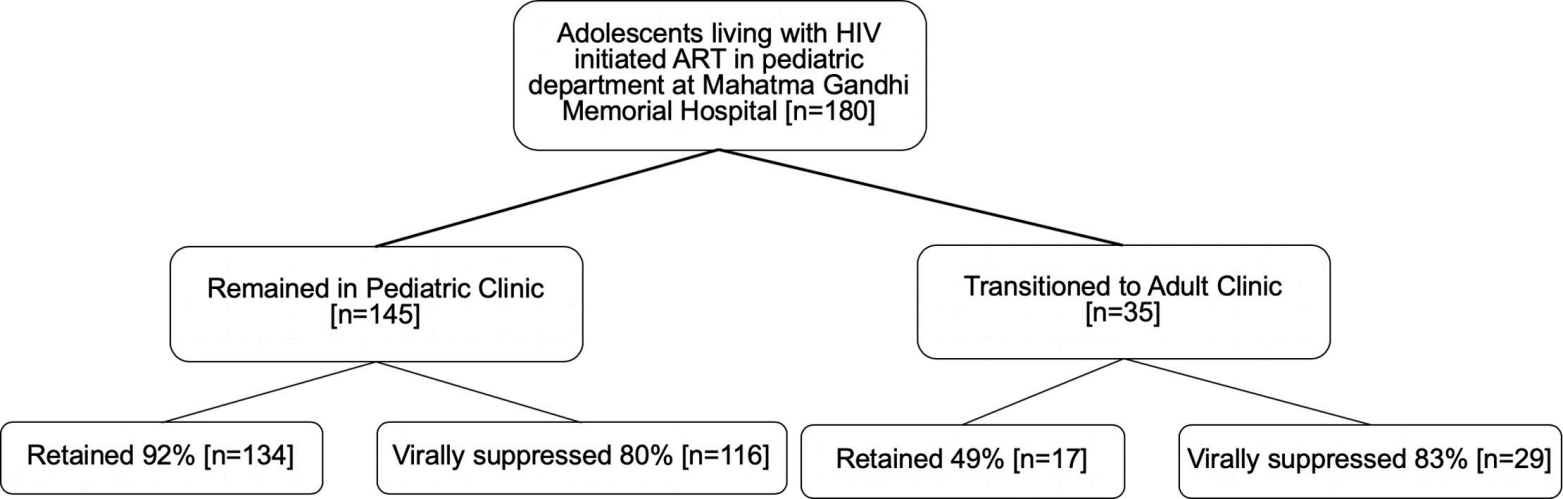
Flow diagram of outcomes among adolescent living with HIV who initiated ART in the pediatric clinic of Mahatma Gandhi Memorial Hospital.

Primary outcomes are shown in **Fig 1 and Table 2**. Adolescents who transitioned to the adult clinic had a lower rate of retention in care (49%; 17/35) compared to adolescents remaining in the pediatric clinic (92%; 134/145; p = <0.001). In the logistic regression model (**Table 3**), retention in care remained lower for adolescents who transitioned to adult care (ARR 0.59; 95%CI 0.43–0.82; p = 0.001) compared to those who remained in pediatric care adjusting for sex, ART regimen, and pre-ART CD4. One year after transition to adult care, there was no difference in viral suppression among adolescents who remained in the pediatric clinic (80%; 116/145) compared to adolescents who transferred to the adult clinic (83%; 29/35) (p = 0.82). Similarly, no association was seen between adolescents who transitioned to the adult clinic compared to adolescents remaining in pediatric care and viral suppression in the multivariable regression model (ARR = 1.06, 95%CI 0.89–1.26; p = 0.53) when adjusting for sex, ART regimen line, and pre-ART CD4.

**Table 2:** Primary outcomes at short-term (age 13) and long-term (most recent results).

Outcomes	Pediatric clinic (n-145)	Transitioned to adult clinic (n = 35)	Crude relative risk (95%CI)	Crude risk difference (95%CI)
**Short-term outcomes**				
** Retained in care at age 13 (n)**	134 (92%)	17 (49%)	0.5 (0.4, 0.7)	-0.5 (-0.6,-0.3)
** Virally suppressed at age 13 (n)**	116 (80%)	29 (83%)	1.0 (0.9, 1.2)	0.1 (-0.1, 0.2)
** Missing viral load at age 13 (n)**	11 (8%)	1 (3%)		
**Long-term outcomes**				
** Virally suppressed at most recent results**	102 (78)	23 (74)	0.9 (0.7, 1.2)	-0,1 (-0.2, 0.1)
** Missing viral load at most recent results**	19 (13%)	5 (14%)		
** Deaths**	3 (2%)	0 (0%)		

**Table 3:** Multivariable analysis of retention in care and short-term (at age 13) and long-term (at most recent results) viral suppression adjusted for sex, ART regimen, and pre-ART CD4.

	Retention in care		Viral suppression at age 13		Viral suppression at most recent results		Combined retention and viral suppression	
**Covariate**	Adjusted risk ratio (95% CI)	p-value	Adjusted risk ratio (95% CI)	p-value	Adjusted risk ratio (95% CI)	p-value	Adjusted risk ratio (95% CI)	p-value
**Sex**	0.98 (0.87, 1.10)	0.69	0.89 (0.77, 1.03)	0.12	0.95 (0.78, 1.17)	0.64	0.92 (0.76, 1.11)	0.38
**Firstline ART**	0.98 (0.84, 1.15)	0.80	1.49 (1.07, 2.08)	0.02	1.59 (1.03, 2.45)	0.035	1.44(0.98, 2.11)	0.062
Pre-ART CD4*	0.99 (0.99, 1.01)	0.37	0.99 (0.98, 1.01)	0.50	0.99 (0.98, 1.01)	0.75	0.99 (0.98, 1.01)	0.42
**Adult clinic vs Pediatric**	0.59 (0.43, 0.82)	0.001	1.06 (0.89, 1.26)	0.53	0.96 (0.73, 1.27)	0.79	0.57 (0.37, 0.87)	0.008

*for each 50 cells/uL

### Long-term outcomes

There was no difference in viral suppression evaluating the most recent viral load results for adolescents who transitioned to adult care (74%; 26/35) compared to those remaining in pediatric care (78%; 113/145) or in the multivariable regression model (ARR = 0.96, 95% CI 0.73–1.27; p = 0.79) as indicated in **Table 3**. Performing a sensitivity analysis excluding missing viral loads did not change the primary outcomes. Using a composite outcome, adolescents who transitioned to adult care were less likely to be retained in care and virally suppressed compared to adolescents remaining in pediatric care when adjusting for **sex**, ART regimen, and pre-ART CD4 (ARR = 0.57, 95%CI 0.37–0.87; p = 0.008) as indicated in **Table 3**.

### Survival analysis

**Fig 2:**
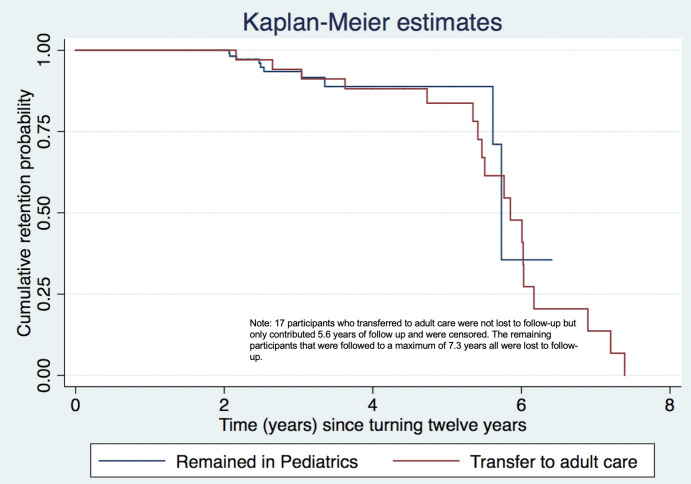
Kaplan-Meier estimate for time to disengagement in care among adolescents living with perinatal HIV in pediatric or adult care.

Overall, the median follow up time was 2.9 years (IQR 2.1–3.9). For adolescents in the pediatric clinic the median follow up time was and 2.6 years (IQR 2.1–3.3) and 5.1 years (IQR 3.9–5.8) for adolescents in the adult clinic. No difference was seen in the adjusted hazard of time to loss to follow-up comparing adolescents who transitioned to adult care and those remaining in pediatric care (AHR 1.8; 95% CI 0.7–4.6; p = 0.26) adjusting for sex, ART regimen and pre-ART CD4. The Kaplan-Meier estimate for time to disengagement in care is indicated in **Fig 2**. The unadjusted hazard ratios are located in the **S1 Table**.

## Discussion

In this hypothesis-generating cohort of adolescents living with perinatally-acquired HIV who were evaluated in a quasiexperimental design, adolescents who remained in pediatric care had higher rates of retention in care compared to those who transitioned to adult care (92% versus 49%; p<0.001). Both groups achieved relatively high rates of viral suppression (80% and 83%, respectively; p = 0.82). While this finding is consistent with the high retention among participants in pediatric care, it is somewhat surprising among those in adult care and likely reflects intermittent engagement in care. The ability to maintain viral suppression without consistent care is questionable; however, retention in care was based on pharmacy refill or missed visits which occur monthly while viral load monitoring occurred annually. Therefore, it is possible to be virally suppressed at last analysis but not retained in care. This situation reflects the intermittent engagement of adolescents. Further research is therefore needed to determine long-term outcomes among adolescents transitioning to adult care, particularly given the high rates of loss to follow-up and mortality generally seen with this population.

In other settings, transition to adult care for perinatally HIV-infected youth has had adverse effects on retention in care, viral suppression, and mortality. An evaluation of transition to adult care for perinatally HIV-infected youth in New York found higher rates of retention in care and viral suppression after transition, but saw mortality increase nearly 25-fold in the first year after transition [16]. In a cohort from Maryland, 60% of perinatally HIV-infected youth were retained in care one year after transition and only 38% were virally suppressed [23]. In a cohort from the Netherlands, virologic failure increased four-fold during the first year after transition of adolescents living with HIV to adult care [15]. These adolescents were significantly less likely to have viral suppression compared to adolescents remaining in the pediatric clinic. An analysis of transition among adolescents living with HIV in the United Kingdom found similar low levels of viral suppression among adolescents before transition (53%) and after transition (48%) [24]. These studies have all occurred in resource-rich countries which may not be applicable to South Africa; however, there is an absence of reported transition outcomes from low- and middle-income settings in the literature.

Optimal timing of transition to adult care is not known. In European studies of adolescents living with HIV, transition occurred between the ages of 17 and 18 years, while in the United States, the median age at transition of between 21 and 24 years [17, 25]. A US-based cohort found that adolescents who transitioned to adult care at older ages had greater satisfaction with their provider and clinic than those who transitioned at a younger age [26]. Adolescents in our South African cohort transitioned to adult care did so at the young age of 12 years old, as is common practice in many government clinics in South Africa [27]. However, Davies et al found that South African adolescents in Cape Town who transferred care at an older age (15–19 years) had higher risk of virologic failure compared to adolescents who transferred at a younger age (10–14 years) [19]. An analysis of the IdDEA cohort in sub-Saharan Africa found that the biggest predictor of being retained in care post transition among age groups of 16, 18, 20 and 22 years was gaps in care the year prior to transition [28]. Neurocognitive, developmental, and social differences among perinatally-HIV infected adolescents makes age-based transition problematic [29, 30]. Given the number of barriers to successful transition, transition readiness assessments could assist clinicians in determining when adolescents should transition to adult care rather than based on age alone [31]. Transition readiness assessments for chronic illnesses in other settings have been used to assist with transition, but have not been used in sub-Saharan Africa [32, 33]. Further research in this area is needed.

Transition to adult care can also be influenced by structural factors. For instance, the transition often involves a change in healthcare providers and a change of healthcare facilities that can negatively impact care [25, 27]. A few studies have evaluated the transfer of children and adolescents to alternate clinics to decongest tertiary care facilities in South Africa. Teasdale et al found that only 67% of children successfully transferred and an additional 21% were lost to follow-up after successful transfer [34]. On the other hand, Ramirez-Avila et al found that 88% of adolescents successfully transferred to government clinics after the closure of a Presidents Emergency Plan for AIDS Relief funded clinic, although notably 48% linked to unintended clinics. These findings suggest that adolescents may be going to clinics that are more convenient resulting in silent transfers and intermittent engagement in care. In neither of these studies were adolescents or providers prepared or trained for transition to adult care. These studies highlight the difficulties in the care for adolescents living with HIV who transfer care facilities often without preparation of the adolescents or the receiving clinicians.

The higher rates of retention in care while being cared for in pediatric care compared to adult care may reflect unique features of the pediatric setting. For instance, adolescents may have felt connected to clinical staff; notably, there is only one pediatric provider. In addition, the unstructured peer group meeting likely provides additional support for retention in care. However, despite higher rates of retention than adult clinics, viral suppression rates and retention in care rates among adolescents cared for in pediatric care still remained below the 90% goals set by the Joint United Nations Programme on HIV/AIDS [35]. A recent study in similar context showed improved retention in care and viral suppression among adolescents living with HIV with the implementation of specific adolescent-friendly services [36]. Adolescent-friendly services could provide a mechanism to assess modifiable factors such as transition readiness, preparation of adolescents and caregivers for transition, and improve communication with healthcare providers to improve the transition process.

This study has some limitations. It was a non-randomized, quasiexperimental design; therefore, unmeasured differences in the adolescents in the adult and pediatric clinics could affect the primary outcomes. In addition, since this analysis involved retrospective review, we could not evaluate several factors that have been shown to influence care outcomes in this population, including knowledge of HIV status, connection to clinic, peer support, transportation, or schooling [37–40]. In addition, due to different frequencies of routine viral load monitoring in the adult and pediatric clinics, we could not conduct an unbiased analysis of time to virologic failure. However, by including the natural experiment, we were able to adjust for some confounding by assignment as all of the adolescents transitioned at age 12. In addition, the adolescents had the same age, ART regimen, follow-up time and were cared for in the same facility by the same physicians using the same national ART guidelines. Since we used the national laboratory database to identify silent transfers, adolescents who were in care, but their data was not recorded would be misclassified as lost to follow-up. The study also had a low sample size resulting in low power to detect small differences in viral suppression. Larger studies may find statistical differences in viral suppression rates for adolescents cared for in different care models. Finally, transitioning to adult care at age 12 years is common in South African government clinics, but may not be generalizable to other settings where transition occurs at older ages.

## Conclusion

Our findings suggest that adolescents living with perinatally acquired HIV in this setting had higher retention in care when cared for in pediatric clinics compared to adult clinics although viral suppression and time to disengagement did not differ among the groups. Given instability of care engagement among those transferred to adult clinics, longer term monitoring of viral suppression is needed. Further research is also required to determine factors that contribute to transition readiness for adolescents living with HIV and if adolescent-friendly services may assist in the transition process. Now is a critical time for research in adolescent transition care and much is needed to advance clinical care for adolescents living with HIV.

## Supporting information

S1 Fig Cumulative number of adolescents transitioning to adult clinic vs. remaining in pediatric clinic by year they turned 12 years of age. (TIF)

S1 Table Unadjusted hazard ratios for time to loss to follow-up. (DOCX)

S1 Data (XLSX)
