# Supplementary figures and images for: Transition from pediatric to adult care for adolescents living with HIV in South Africa: A natural experiment and survival analysis
Source: PLoS One. 2020 Oct 27;15(10):e0240918. doi: 10.1371/journal.pone.0240918 (PMC7591089; doi:10.1371/journal.pone.0240918)

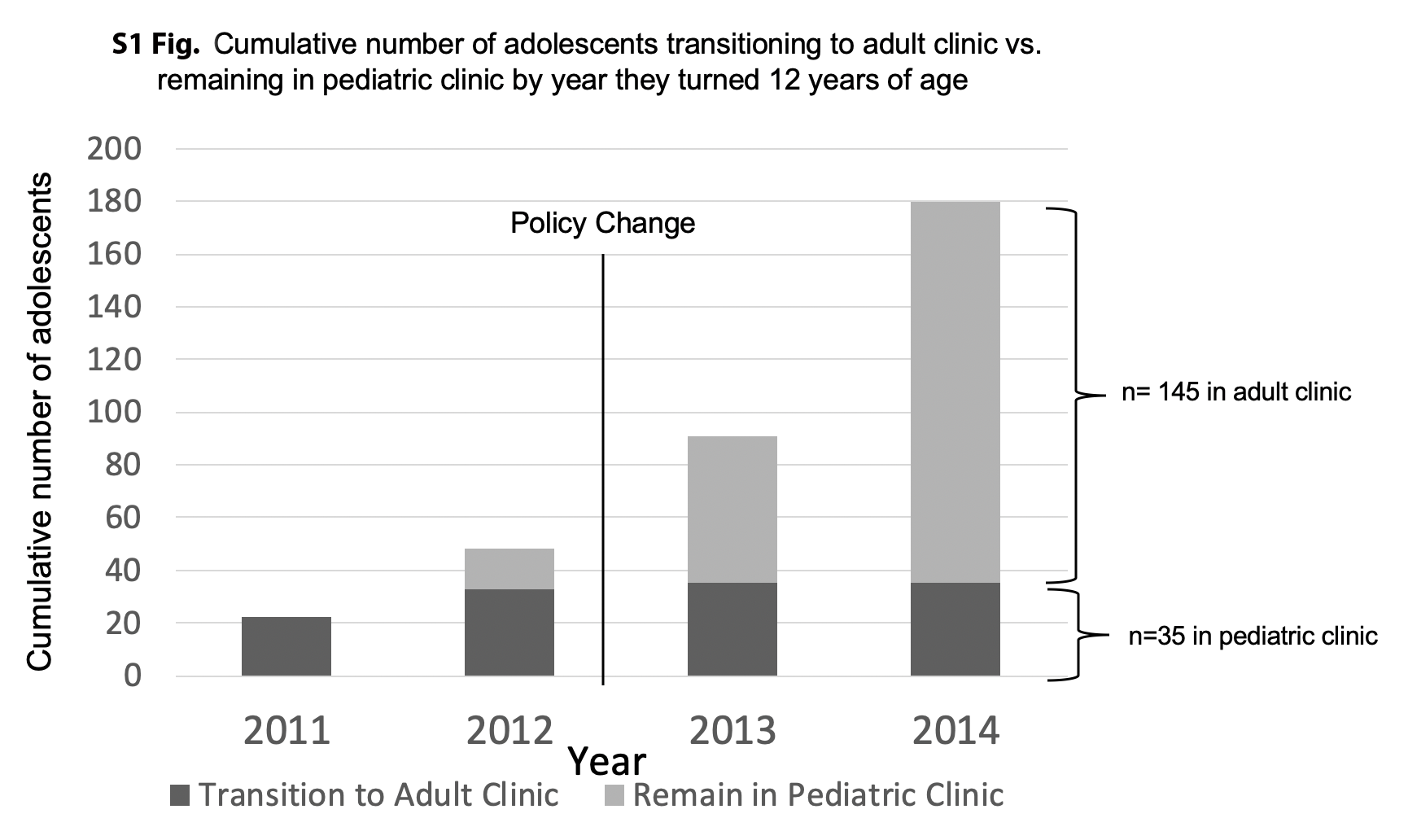

Supplement: S1 Fig — (TIF) [file pone.0240918.s001.tif]
