# Supplementary material for: Transition from pediatric to adult care for adolescents living with HIV in South Africa: A natural experiment and survival analysis
Source: PLoS One. 2020 Oct 27;15(10):e0240918. doi: 10.1371/journal.pone.0240918 (PMC7591089; doi:10.1371/journal.pone.0240918)
Supplement: S1 Table — (DOCX) [file pone.0240918.s002.docx]

S1 Table. Unadjusted hazard ratios for time to loss to follow-up

|  |  | Unadjusted Hazard Ratios | | |
| --- | --- | --- | --- | --- |
| Covariate | Unadjusted hazard ratio (95% CI) | |  | p-value |
| Sex | 0.53 (0.25, 1.11) | |  | 0.09 |
| Firstline ART | 0.85 (0.32, 2.26) | |  | 0.74 |
| Pre-ART CD4 (for each 50 cells/uL) | 1.05 (0.97, 1.13) | |  | 0.20 |
| History of Tuberculosis treatment | 1.03 (0.49, 2.17) | |  | 0.94 |
| Adult vs pediatric clinic | 1.20 (0.50, 2.90) | |  |  |
